# The choice of reference chart affects the strength of the association between malaria in pregnancy and small for gestational age: an individual participant data meta-analysis comparing the Intergrowth-21 with a Tanzanian birthweight chart

**DOI:** 10.1186/s12936-022-04307-2

**Published:** 2022-10-12

**Authors:** George Mtove, Daniel T. R. Minja, Omari Abdul, Samwel Gesase, Kenneth Maleta, Titus H. Divala, Noel Patson, Ulla Ashorn, Miriam K. Laufer, Mwayiwawo Madanitsa, Per Ashorn, Don Mathanga, Jobiba Chinkhumba, Julie R. Gutman, Feiko O. ter Kuile, Sofie Lykke Møller, Ib C. Bygbjerg, Michael Alifrangis, Thor Theander, John P. A. Lusingu, Christentze Schmiegelow

**Affiliations:** 1 Tanga Medical Research Centre, National Institute for Medical Research, P. O. Box, 210, Tanga, Tanzania; 2 Kamuzu University of Health Sciences, Blantyre, Malawi; 3 Tampere Center for Child, Adolescent and Maternal Health Research, Faculty of Medicine and Life Sciences, University of Tampere, Tampere, Finland; 4 University of Maryland School of Medicine, Baltimore, USA; 5 Malawi University of Science and Technology, Thyolo, Malawi; 6 Faculty of Medicine and Health Technology, Center for Child, Adolescent, and Maternal Health Research, Tampere University, Tampere, Finland; 7 Department of Paediatrics, Tampere University Hospital, Tampere, Finland; 8 Malaria Branch, Division of Parasitic Diseases and Malaria, Center for Global Health, US Centers for Diseases Control and Prevention, Atlanta, GA USA; 9 Department of Clinical Sciences, Liverpool School of Tropical Medicine, Liverpool, UK; 10 Section of Global Health, Department of Public Health, University of Copenhagen, Copenhagen, Denmark; 11 Centre for Medical Parasitology, Department of Immunology and Microbiology, University of Copenhagen, Copenhagen, Denmark; 12 Department of Infectious Diseases, Copenhagen University Hospital (Rigshospitalet), Copenhagen, Denmark

**Keywords:** Malaria in pregnancy, Birthweight, Reference chart, Individual participant data meta-analysis

## Abstract

**Background:**

The prevalence of small for gestational age (SGA) may vary depending on the chosen weight-for-gestational-age reference chart. An individual participant data meta-analysis was conducted to assess the implications of using a local reference (STOPPAM) instead of a universal reference (Intergrowth-21) on the association between malaria in pregnancy and SGA.

**Methods:**

Individual participant data of 6,236 newborns were pooled from seven conveniently identified studies conducted in Tanzania and Malawi from 2003–2018 with data on malaria in pregnancy, birthweight, and ultrasound estimated gestational age. Mixed-effects regression models were used to compare the association between malaria in pregnancy and SGA when using the STOPPAM and the Intergrowth-21 references, respectively.

**Results:**

The 10th percentile for birthweights-for-gestational age was lower for STOPPAM than for Intergrowth-21, leading to a prevalence of SGA_STOPPAM_ of 14.2% and SGA_IG21_ of 18.0%, p < 0.001. The association between malaria in pregnancy and SGA was stronger for STOPPAM (adjusted odds ratio (aOR) 1.30 [1.09–1.56], p < 0.01) than for Intergrowth-21 (aOR 1.19 [1.00–1.40], p = 0.04), particularly among paucigravidae (SGA_STOPPAM_ aOR 1.36 [1.09–1.71], p < 0.01 vs SGA_IG21_ aOR 1.21 [0.97–1.50], p = 0.08).

**Conclusions:**

The prevalence of SGA may be overestimated and the impact of malaria in pregnancy underestimated when using Intergrowth-21. Comparing local reference charts to global references when assessing and interpreting the impact of malaria in pregnancy may be appropriate.

**Supplementary Information:**

The online version contains supplementary material available at 10.1186/s12936-022-04307-2.

## Background

The World Health Organization (WHO) estimated that 11.6 million pregnant women in sub-Saharan Africa were exposed to malaria in 2020 [1]. Malaria in pregnancy (MIP) is associated with adverse pregnancy outcomes, such as small for gestational age (SGA) [2–4], with the worst consequences occurring after infection in the first and second trimesters, particularly among paucigravidae [2, 4, 5].

Small for gestational age is defined as a birthweight below a pre-defined cut-off for a specific sex and gestational age (GA), often the 10th percentile [6, 7]. Decreasing birthweight is associated with higher neonatal morbidity and mortality [8–12] as well as long-term complications, including cardio-metabolic diseases [13–15]. Thus, SGA is a commonly used outcome in clinical trials on interventions to prevent MIP. The choice of reference chart is important for an accurate diagnosis of SGA [7, 16, 17]. In randomized clinical trials evaluating the efficacy of malaria interventions for improving pregnancy outcomes, misclassification of adequate for gestational age (AGA) as SGA and vice versa may dilute the observed treatment effect.

The Intergrowth-21 birthweight chart (IG21) is a universally applicable reference developed based on the assumptions that in healthy (low-risk) pregnancies, all fetuses achieve a similar growth potential, irrespective of ethnic or geographical differences, and that maternal and paternal anthropometric characteristics do not influence birthweight [18]. However, the WHO multicountry [19] and the National Institute of Child Health and Development fetal growth [16] studies found significant ethnic and geographic differences in newborn’s size. Furthermore, several other studies have indicated that local weight references surpass IG21 in terms of diagnostic accuracy of SGA and its association with poor pregnancy outcomes [20–27]. This suggests that the IG21 may not be appropriate for identifying SGA in all settings, as recently concluded by the International Federation of Gynaecology and Obstetrics (FIGO) [17].

In 2011, a local Tanzanian reference chart (STOPPAM) was developed using data from 583 healthy newborns [28] as a hybrid of fetal weights (until 38 weeks) and birthweights. Thus, the STOPPAM reference would represent the newborns’ potential size while taking the ethnic/geographical differences into account. STOPPAM is the largest chart in East Africa using ultrasound to estimate GA and fetal weight while excluding all newborns at high risk of poor fetal growth. Furthermore, the criteria used to define a healthy pregnancy were quite similar to IG21 (Additional file 1: Table S1) despite the differences in the construction of the two reference charts [29–34]. Other references from sub-Saharan Africa either did not use ultrasound for GA estimation and included malaria positive women [35] or were smaller than STOPPAM [36, 37].

In this individual participant data meta-analysis, the performance of the IG21 was compared to the STOPPAM reference in estimating the association between MIP and SGA.

## Methods

### Study design

This was a meta-analysis of individual participant data from studies in Tanzania and Malawi. The primary outcome was SGA defined as birthweight < 10th percentile based on IG21 or STOPPAM sex-specific references while the primary exposure was MIP.

The study hypothesized that IG21 would overestimate the prevalence of SGA and ultimately weaken the strength of the association between MIP and SGA.

### Search strategy and inclusion criteria

A literature search was performed through PubMed, Medline, the Cochrane Library, and EMBASE. The search terms were (“malaria in pregnancy” OR “plasmodium malaria”) AND (SGA OR birthweight OR “birth weight”). In addition, personal communication was made for unpublished studies identified through existing research network. The inclusion criteria were pregnancy-related studies from East and Central Africa with data on GA estimated using ultrasound, birthweight measured within 24 h post-delivery or adjusted if measured > 24 h, newborns’ sex, and MIP.

### Study selection and data gathering

**Fig. 1:**
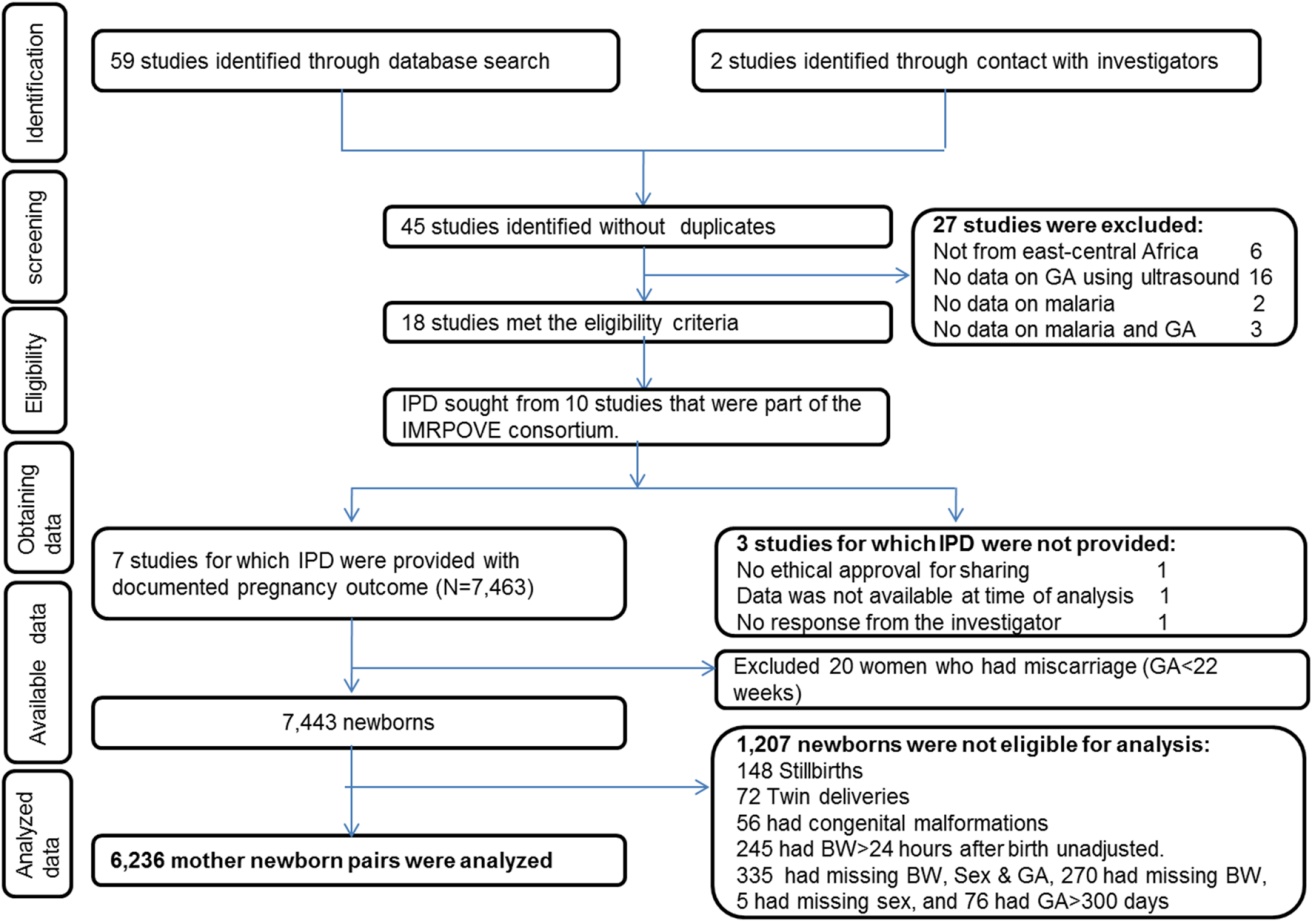
Flow diagram for obtaining individual patient data (IPD). ANC: antenatal care, BW: birth weight, GA: gestational age, IMPROVE: Improving pregnancy outcome using intermittent preventive treatment of malaria in pregnancy

Two authors (GM and CS) selected potentially relevant studies according to the eligibility criteria after reviewing the abstracts or full texts (Fig. 1). Then, raw data were conveniently sought for ten studies in which the authors were part of a consortium working on improving pregnancy outcomes using intermittent preventive treatment of malaria in pregnancy (IPTp) (https://www.improve-consortium.org/). Eight authors agreed to collaborate, and seven of them shared the individual participant data (Fig. 1).

### Study population

The meta-analysis included all live-born singleton newborns without congenital malformation, and with birthweight measured within 24 h of birth or birthweights measured > 24 h if the original study teams had already adjusted them [38, 39], known sex and GA estimated using ultrasound. Exclusion criteria were: stillbirth, multiple pregnancies, congenital malformation, unadjusted weights measured > 24 h post-delivery, and missing data on birthweight, sex, and/or GA.

### Assessment of the risk of bias

The quality of each study was evaluated by one author (GM) using the Cochrane tool for individually randomized trials [40] and the Newcastle–Ottawa scale for cohort studies [41], and focused on how data on birthweight and MIP were obtained.

### Statistical analysis

Analysis was done using Stata software, version 16 (Stata Corp, Texas, USA). Continuous variables were described as a mean with standard deviation (SD) or a median with interquartile range (IQR). Categorical variables were summarized as proportions with 95% confidence intervals (CI). The percentiles (10th, 50th, and 90th), GA-specific birthweight z-scores, and the prevalence of SGA were compared for the IG21 and STOPPAM references. In addition, SGA was stratified as low birthweight (LBW < 2.5 kg) and normal birthweight (NBW ≥ 2.5 kg).

MIP was defined as any positive test at any point during pregnancy (microscopy, malaria rapid diagnostic test (RDT), polymerase chain reaction (PCR), or placental histology). In all analyses, the malaria-negative control group was defined as never having had malaria detected on any test.

A one-stage approach was used to combine the individual participant data using a mixed-effects regression model. The unadjusted and adjusted odds ratios (uOR and aOR) for SGA in relation to MIP were calculated using this model. The overall effect was obtained from the preceding mixed-effects regression and the study-specific effects were obtained by logistic regression. This was done separately for both references, SGA_STOPPAM_ and SGA_IG21_, and the results graphically presented as forest plots.

The main model compared SGA and MIP. This was further stratified by gravidity (paucigravidae (1st and 2nd pregnancy) vs multigravidae) and the time of infection (malaria in the 1st and 2nd, all trimesters, or 3rd trimester only vs malaria-negative). In sensitivity analyses, data were restricted to (a) only HIV-negative women-newborn pairs, (b) exclusion of sub-patent infections (PCR positive, microscopy negative), past infections (RDT positive, microscopy negative), or only positive placental histology (negative microscopy, RDT and PCR), (c) exclusion of studies that only used RDT [38] or only used PCR to detect malaria [42], (d) exclusion of a study only testing symptomatic cases at antenatal visits (Gutman et al., unpublished), (e) exclusion of newborns with adjusted weight measured > 24 h post-delivery, (f) exclusion of newborns with GA-delivery ≤ 38 weeks as the STOPPAM reference was generated based on fetal weights in addition to birthweight until this GA, (g) only SGA_IG21_-AGA_STOPPAM,_ (h) only SGA_STOPPAM_-AGA_IG21_.

In addition, the inclusion criteria used in the STOPPAM and IG21 studies were applied to the cohort [28, 43] and the prevalence of SGA in the “low-risk” cohort was re-evaluated with the assumption that a SGA prevalence of 10% would indicate the reference as representative.

Potential confounders were tested in univariate analysis using a mixed-effects regression model and included GA at enrolment and delivery, gravidity, maternal age, hemoglobin level, mid-upper arm circumference, and body mass index (BMI) at enrolment, utilization of IPTp, treated bed net, and iron plus folic-acid supplements, the number of antenatal visits, and syphilis or HIV-positivity. Variables with p-values < 0.2 in the univariate analysis were included in the multivariate model, and a step-wise backward elimination approach was used to obtain the final model, retaining confounders with a p < 0.1. A p-value < 0.05 was considered statistically significant.

I^2^ statistics could not be estimated in the one-stage approach because residual variability is not reported under mixed-effects regression with binary outcomes [44]. Hence, the parameters were fitted as random effects in the model to account for between-study variations [44]. Furthermore, a two-stage IPD meta-analysis was performed to assess heterogeneity between studies using I^2^ statistics and to assess the robustness of the analyses.

### Publication bias

A funnel plot was used to evaluate publication bias. The trim and fill method and contour enhancement funnel plot were used to determine whether the asymmetry in the forest plot was due to a small study effect or factors other than publication bias.

## Results

### Search results

The seven studies covered 7,443 newborns (Fig. 1). Of these, 521 were excluded due to: stillbirth (n = 148), congenital malformation (n = 56), twin pregnancy (n = 72), or unadjusted birthweight measured > 24 h post-delivery (n = 245). Of the 6922 remaining newborns, 610 were missing birthweight, sex, and/or GA, and 76 had GA > 42 + 6 weeks, which is above the range of the two references. Hence, 6,236 mother-newborn pairs were analysed.

### Study description

Among the included studies, two were prospective cohorts [28, 45], and five were randomized trials, including one partially blinded [46] and four open-label [38, 39, 42] (Gutman et al., unpublished) (Table 1). Women were enrolled from the 2nd trimester in four studies [39, 42, 46] (Gutman et al., unpublished) and from the 1st trimester in three studies [28, 38, 45]. All studies had malaria related objectives except for one study on lipid-based nutrient supplements [38]. All women in this study received routine IPTp with sulfadoxine-pyrimethamine. Malaria testing strategies varied between studies: only RDT [38]; only PCR [42]; microscopy and PCR [46]; RDT, microscopy and PCR [28, 45]; RDT, microscopy, PCR and placental histology [39]; or PCR, microscopy and RDT at enrolment and delivery visits, but during antenatal visits only symptomatic women were tested (Gutman et al., unpublished). Three studies enrolled only HIV-seronegative women [39, 42] (Gutman et al., unpublished). The remaining four included HIV-positive women as well [28, 38, 45, 46].

**Table 1:** Characteristics of the analyzed mother-newborn pair

		Luntamo et al. [46]	Schmiegelow et al. [28]	Madanitsa et al. [39]	Ashorn et al. [38]	Divala et al. [42]		Moeller et al. [45]		Gutman et al. *Unpublished*	All
Maternal^a^											
Study period		2003–2006	2008–2010	2011–2013	2011–2012	2012–2014		2014–2016		2017–2018	2003–2018
Sample size		1,173	752	1,607	1,109	747		375		473	6,236
Study design		RCT	Prospective cohort	RCT	RCT	RCT		Prospective cohort		RCT	–
Study location		Malawi	Tanzania	Malawi	Malawi	Malawi		Tanzania		Malawi	Tanzania & Malawi
Inclusion criteria		No severe illness, 14–26 weeks	All Consenting women ≤ 24 weeks	HIV neg, Hb > 7 g/dl, no risk factor, 16–28 weeks	≥ 15yrs, no risk factor, 14–20 weeks	HIV negative, paucigravidae, 15–28 weeks		All Consenting women, 4–28 weeks		≥ 16yrs, HIV negative, no risk factor, ≤ 28 weeks	–
GA estimation method		Transabdominal ultrasound (CRL or HC)	Transabdominal ultrasound (CRL or HC)	Transabdominal ultrasound (HC)	Transabdominal ultrasound (BPD, AC, FL)	Transabdominal ultrasound (HC)		Transabdominal ultrasound (CRL or HC)		Transabdominal ultrasound (HC)	–
GA at entry (wks)		20 (18–23)	19 (15–21)	21 (19–23)	17 (15–19)	21 (22–24)		10 (7–13)		20 (18–22)	17 (19–22)
Trimester at enrolment											
First (≤ 13 weeks)		0 (0.0%)	85 (11.3%)	0 (0.0%)	30 (2.7%)	0 (0.0%)		284 (75.7%)		0 (0.0%)	399 (6.4%)
Early second (14-21 weeks)		815 (69.5%)	554 (73.7%)	1,064 (66.2%)	1,079 (97.3%)	386 (51.3%)		81 (21.6%)		329 (70.0%)	4,251 (68.2%)
Late second (22-27 weeks)		358 (30.5%)	113 (15.0%)	444 (27.2%)	0 (0.0%)	329 (44.0%)		8 (2.1%)		127 (26.4%)	1,433 (23.0%)
Third (≥ 28 weeks)		0 (0.0%)	0 (0.0%)	99 (6.2%)	0 (0.0%)	35 (4.7%)		2 (0.5%)		17 (3.4%)	153 (2.5%)
Follow up schedule		Four-weekly intervals until 36 weeks and weekly thereafter	Enrolment, at week 26–28, 30–32, 36–38, sick and delivery visits	Every four to six weeks until sick and delivery visits	Enrolment, at week 32, 36 and delivery	At least once every four weeks until delivery		Enrolment, at week 11–14, 20–22, 26–28, 32–34, 37–39, sick and delivery visits		Four weekly until delivery and sick visit	–
Type of weighing scale		Spring scale ((Super Samson, Salter Brecknell, 50 g) or digital (SECA 834, Chasmors Ltd, 10 g)	Digital scale (ADE, 10 g) or Fazzini spring (50 g) on few	Not specified	SECA 381 baby scale, Seca GmbH & Co)	Not specified		Digital scale (precision, 5–10 g; M107600, ADE)		Digital scale	–
Adjusted BW > 24 h after birth		0 (0.0%)	0 (0.0%)	76 (4.7%)	338 (30.5%)	0 (0.0%)		0 (0.0%)		0 (0.0%)	414 (6.6%)
Type of malaria test	Microscopy or PCR		Microscopy or mRDT (Parascreen, Paracheck or ParaHIT) PCR on all mRDT positive	Microscopy or mRDT(First Response Malaria pLDH/HRP2 Combo Test), PCR and placental histology	mRDT (Clearview Malaria Combo; British Biocell International Ltd)		PCR	Microscopy, mRDT(ParaHIT or CareStart) and PCR^b^		Microscopy, mRDT (Paracheck) and PCR	–
Age (years)	24 (20–29)		26 (22–31)	21 (18–26)	25 (20–29)		21 (19–23)	27 (22–34)		23 (19–29)	23 (19–28)
MUAC (cm)^c^	25.2 (± 2.1)		26.1 (± 2.9)	ND	26.3 (± 2.5)		ND	28.2 (± 3.8)		26.3 (± 3.2)	26.1 (± 2.8)
Weight (Kg) ^c^	52.4 (6.3)		55.0 (± 10.2)	55.2 (± 5.4)	54.1 (± 8.0)		58.9 (± 8.1)	57.5 (± 11.6)		58.5 (± 10.0)	55.9 (± 8.5)
Height (cm) ^c^	155.0 (± 5.5)		157.5 (± 5.8)	154.0 (± 5.0)	156.1 (± 5.6)		157.3 (± 5.7)	155.4 (± 5.8)		157.3 (± 6.2)	156.0 (± 5.7)
BMI (kg/m2)^c^	21.8 (± 2.2)		22.2 (± 3.6)	23.3 (± 2.9)	22.0 (± 2.7)		23.8 (± 3.0)	23.7 (± 4.2)		23.7 (± 4.1)	22.8 (± 3.2)
Gravidity:											
Paucigravidae	477 (40.7%)		345 (45.9%)	993 (61.9%)	431 (38.9%)		746 (100.0)	106 (28.3%)		266 (56.5%)	3,364 (54.0%)
Multigravidae	696 (59.3%)		407 (54.1%)	612 (38.1%)	676 (61.1%)		0 (0.0)	269 (71.7%)		205 (43.5%)	2,865 (45.9%)
HIV status:											
Positive	144 (12.3%)		39 (5.2%)	NA	147 (13.3%)		NA	10 (2.7%)		NA	340/3,409 (9.9%)
Negative	913 (77.8%)		656 (87.2%)	NA	952 (85.8%)		NA	357 (95.2%)		NA	2,878/3,409(84.4%)
Missing	116 (9.9%)		57 (7.6%)	NA	10 (0.9%)		NA	8 (2.1%)		NA	191/3,409 (2.7%)
Syphilis positive	58 (5.0%)		ND	ND	ND		ND	ND		2 (0.4%)	60 (3.9%)
ITN use	839 (71.5%)		714 (95.0%)	1,605 (99.9%)	1,087 (98.4%)		565 (75.6%)	353 (94.1%)		223 (47.2%)	5,841 (86.1)
# ANC visits	NA		4 (4–5)	4 (3–4)	4 (3–5)		ND	7 (6–9)		4 (4–5)	4 (3–5)
# of IPTp doses	4 (2–4)		2 (2–2)	2 (1–4)	ND		ND	3 (2–4)		ND	2 (2–4)
Iron use	ND		698 (92.6%)	1,802 (99.0%)	ND		ND	370 (98.7%)		ND	2,903 (82.8%)
Ever anaemic	449 (38.3%)		458 (60.9%)	451 (28.1%)	312 (28.1%)		169 (23.5%)	161 (42.9%)		146 (30.9%)	2,146 (34.6%)
Hb (g/dl)^c^											
Enrolment	11.0 (1.9)		10.9 (1.7)	11.0 (1.5)	11.2 (1.6)		11.7 (1.3)	11.6 (1.4)		10.7(1.3)	11.1 (1.5)
Delivery	11.3 (1.8)		10.7 (1.4)	11.8 (1.6)	ND		ND	11.2 (1.5)		11.8 (1.5)	11.4 (1.4)
Overall malaria positivity in pregnancy	168 (14.3%)		52 (6.9%)	1,214 (75.5%)	400 (36.1%)		114 (15.3%)	143 (38.1%)		141 (29.8%)	2,232 (35.8%)
Malaria positivity rate by diagnostic test:											
mRDT	ND		48/752 (6.4%)	337/1,607 (21.0%)	400/1105(36.1%)		ND	142/375(37.9%)		31/473 (6.6%)	958/4,316(22.2%)
PCR	50/ 456 (11.0%)		ND	1,095/1,600(68.4%)	ND		114/746(15.3%)	125/375(33.1%)		127/473(26.9%)	1,511/ 3,650(41.4%)
Slide	136/1,173 (11.6%)		33/752 (4.4%)	553/1,607(34.4%)	ND		ND	90/375(24.0%)		10/465 (2.1%)	822/ 4,372(18.8%)
Placenta histology	ND		ND	400/1,607(24.9%)	ND		ND	ND		ND	400/1607(24.9%)
Newborn^a^											
GA del, wks	39 (38–40)		40 (39–41)	38 (37–40)	40 (39–41)		39 (38–40)	40 (39–41)		39 (37–40)	39 (38–40)
Preterm	113 (9.6%)		23 (3.1%)	259 (16.1%)	56 (5.1%)		75 (10.1%)	17 (4.5%)		67 (14.2%)	616 (9.9%)
Sex = Male	594 (50.6%)		372 (49.2%)	846 (50.2%)	609(48.8%)		369(48.6%)		184(49.1%)	267 (54.1%)	3,093 (49.6%)
BW (gm)^c^	2,967 (± 462)		3,154 (± 472)	2,936 (± 428)	2,981(± 453)		2,910 (± 393)		3,022 (± 461)	2,988 (± 410)	2,980 (± 451)
LBW	109 (9.3%)		47 (6.3%)	173 (10.8%)	140 (12.6%)		91 (12.2%)		39 (10.4%)	40 (8.5%)	639 (10.3%)
SGA_STOPPAM_:											
Malaria	35/168(20.8%)		11/52(21.1%)	176/1,214(14.5%)	88/400(22.0%)		17/114(14.9%)		29/143(20.3%)	20/141(14.2%)	376/2,232(16.8%)
No malaria	150/1,005(14.9%)		67/700(9.6%)	43/393(10.9%)	104/709(14.7%)		90/632(14.2%)		40/232(17.2%)	16/332(4.5%)	510/4,003(12.8%)
Overall	185/1,173(15.8%)		78/752 (10.4%)	219/1,607 (13.6%)	192/1,109(17.3%)		107/747(14.5%)		69/375 (18.4)	36/473 (7.6%)	886/6,235 (14.2)
SGA_IG21_:											
Malaria	35/168 (20.8%)		15/52(28.2%)	187/1,214(15.4%)	113/400(28.3%)		19/114(16.7%)		40/143(28.0%)	24/141(17.0%)	433/2,232(19.4%)
No malaria	177/1,005(17.6%)		114/700(16.3%)	46/393(11.7%)	146/709(20.6%)		120/632(19.0%)		59/232(25.4%)	28/332(8.0%)	690/4,003(17.2%)
Overall	212/1,173(18.2%)		129.752 (17.2%)	233/1,607 (14.5%)	259/1,109(23.4)		140/747(18.7%)		99/375(26.4%)	52/473(11.0%)	1,123/6,235(18.0%)

*RCT:* randomized controlled trial, *CRL:* crown rump length, *HC:* head circumference, *AC:* abdominal circumference, *FL:* femur length, *ND* not done, *NA* not applicable, *MUAC:* mid upper arm circumference, *BMI:* body mass index, *HIV:* Human Immunodeficiency Virus, *ITN:* treated bed nets, *ANC:* antenatal care, *IPTp:* intermittent preventive treatment of malaria in pregnancy, *Hb:* hemoglobin, *GA:* gestational age, *preterm:* GA < 37 weeks, *BW* birthweight, *LBW*: BW < 2.5 kg, *SGA*_*STOPPAM*_: small for gestational age using STOPPAM reference, *SGA*_*IG21*_: SGA using Intergrowth-21 reference, total is < 100% in case of missing data

^a^All results are number (%) or median (Interquartile range) unless stated otherwise

^b^Malaria species diagnostic polymerase chain reaction (PCR) was also done for all mRDT-positive samples and samples for those who were always mRDT negative collected at GA 26–28 weeks or at delivery

^c^mean (standard deviation)

### The quality and characteristics of the included studies

Two randomized trials were graded as being of good quality; the other three were potentially at high risk of bias (Additional file 1: Table S2). The observational studies were considered to have low risk of bias (Additional file 1: Table S3).

The maternal and newborns’ characteristics are presented in Table 1. The median (IQR) maternal age was 23 (19–28) years, and > 50% of the women were paucigravidae. Among the four studies including women with HIV, 10.0% (340/3,409) were HIV-seropositive. The cumulative malaria incidence varied from 6.9% to 75.5%, with an average of 35.6% for all studies. The median (IQR) GA at delivery was 39 + 0 (38 + 0–40 + 0) weeks, and 9.9% were born preterm. The mean (SD) birthweight was 2978 (451) grams, with 10.3% being LBW (< 2500 g).

### Birthweight percentiles and prevalence of SGA

The weight percentiles differed between the STOPPAM and IG21 references depending on GA (Additional file 2: Fig. S1). The 10th percentiles for the two references converged at a GA of 36 + 2 for boys and 38 + 1 for girls. The IG21 had a lower 10th percentile than the STOPPAM reference at earlier GA, with a mean difference of − 105 g for boys and −136 g for girls at GA 36 + 0. At later GA, the direction shifted, with the 10th percentile for IG21 being higher, resulting in a mean difference of + 133 g for boys and + 123 g for girls at GA 40 + 0 compared to the STOPPAM reference. A similar pattern was also observed for the 50th percentile, whereas the 90th percentile for the IG21 was higher compared to the STOPPAM reference throughout gestation (Additional file 2: Fig. S1). The mean (SD) birthweight z-score was − 0.14 (1.19) for the STOPPAM and − 0.42 (0.99) for IG21 reference (Additional file 3: Fig. S2).

The differences in the 10th percentile were reflected in the prevalence of SGA. Fewer newborns were SGA when using the STOPPAM compared to the IG21 reference (14.2% (95% CI: 13.4–15.1) vs 18.0% (95% CI: 17.1–19.0)), p < 0.001) (Table 2) and the proportion of SGA varied with GA. Preterm, the prevalence of SGA was higher when using the STOPPAM reference (SGA_STOPPAM_ 15.4% (95% CI: 12.6–18.3) vs. SGA_IG21_ 7.0% (95% CI: 5.0–9.0), p < 0.001). All preterm SGA newborns were also LBW (Additional file 1: Table S4). At term, the prevalence was higher when using the IG21 reference (SGA_IG21_ 19.3% (95% CI: 18.2–20.3) vs. SGA_STOPPAM_ 14.1% (95% CI: 13.2–15.0), p < 0.001) (Table 2). The excess proportion of SGA_IG21_ was especially among NBW-term newborns (Additional file 1: Table S4). When applying IG21 and STOPPAM inclusion criteria, SGA_IG21_ were 14.9% and 17.2%, whereas SGA_STOPPAM_ were 9.6% and 11.5%, respectively (Additional file 1: Table S5).

**Table 2:** Comparing the prevalence of small for gestational age among preterm, full term and all infants using the STOPPAM and the Intergrowth-21 references

Preterm (N = 616)				Full term infants (N = 5,620)			All infants (N = 6,236)		
SGA_IG21_	SGA_STOPPAM_, n (%)			SGA_STOPPAM_, n (%)			SGA_STOPPAM_, n (%)		
	Yes	No	Total	Yes	No	Total	Yes	No	Total
Yes	43 (7.0)	0 (0.0)	43 (7.0)	745 (13.3)	336 (6.0)	1,081 (19.3)	788 (12.6)	336 (5.4)	1,124 (18.0)
No	52 (8.4)	521 (84,6)	573 (93.0)	47 (0.8)	4,492 (79.9)	4,539 (80.7)	99 (1.6)	5,013 (80.4)	5,112 (82.0)
Total	95 (15.4)	521 (84.6)	616 (100)	792 (14.1)	4,828 (85.9)	5,620 (100)	887 (14.2)	5,349 (85.8)	6,236 (100)

*SGA*_*STOPPAM*_: small for gestational age (SGA) using STOPPAM reference, *SGA*_*IG21*_: SGA using intergrowth-21 reference, *Preterm:* gestational age (GA) < 37 weeks, *Full term*: GA ≥ 37 weeks

### Association between MIP and SGA

**Fig. 2:**
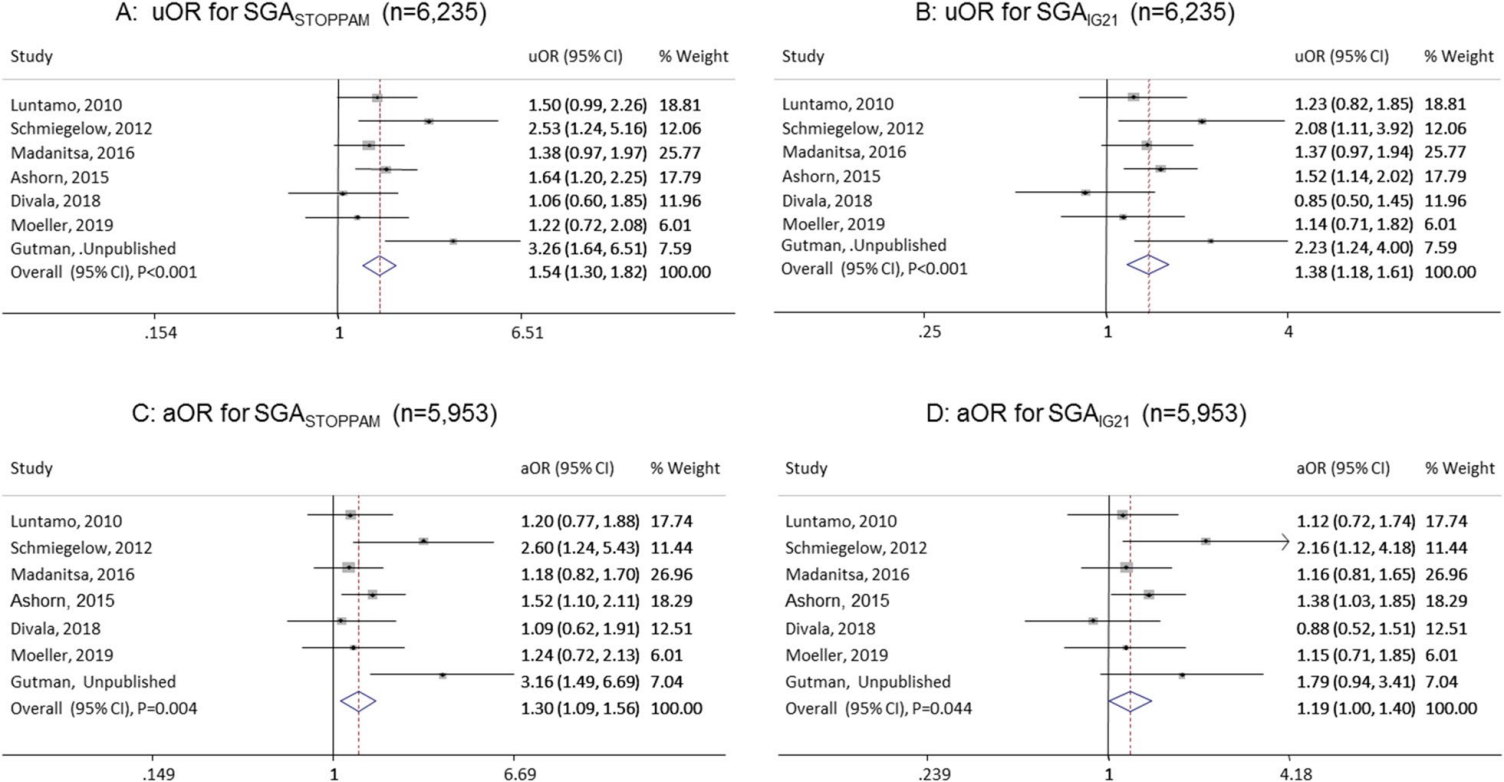
Association between malaria in pregnancy and small for gestational age using the STOPPAM reference (SGA_STOPPAM_) vs. the Intergrowth-21 reference (SGA_IG21_**).** uOR: unadjusted odds ratio in panels **A** and **B**, aOR: adjusted odds ratio in panels **C** and **D** controlling for body mass index, gravidity, gestational age at enrolment, HIV, and hemoglobin level at enrolment. In addition, adjusted for gestational age at delivery for SGA_IG21_, *CI* confidence interval, malaria was defined as positive slide or positive malaria rapid test or positive polymerase chain reaction or positive placenta histology, % Weights are from random effects analysis

The STOPPAM reference was more sensitive than the IG21 reference in detecting an association between MIP and SGA (STOPPAM: uOR 1.54 (95% CI: 1.30–1.82), p < 0.001; aOR 1.30 (1.09–1.56), p = 0.004. IG21: uOR 1.38 (95% CI: 1.18–1.61), p < 0.001; aOR 1.19 (1.00–1.40), p = 0.044) (Fig. 2).

**Fig. 3:**
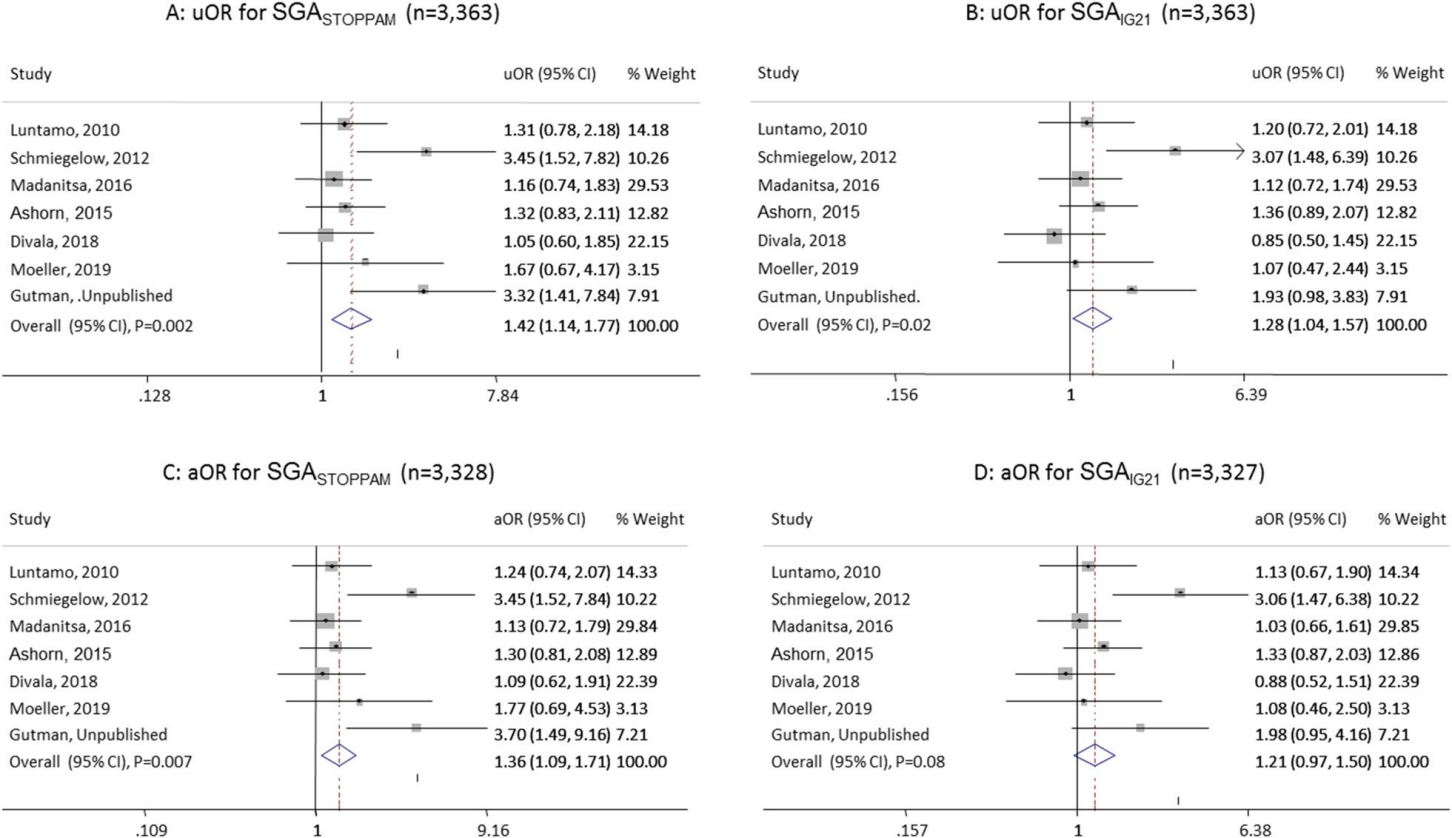
Association between malaria among paucigravidae and small for gestational age using the STOPPAM (SGA_STOPPAM_) vs. the Intergrowth (SGA_IG21_) references, uOR: unadjusted odds ratio in panels **A** and **B**, aOR: adjusted odds ratio in panels **C** and **D** controlling for body mass index and gestational age at enrolment. In addition, adjusted for gestational age at delivery and hemoglobin level at enrolment for SGA_IG21_ hence n = 3,327 due to missing value for hemoglobin level, *CI* confidence intervals, % Weights are from random effects analysis

When only including newborns of paucigravidae women, the results were robust, with slightly higher uOR for SGA_STOPPAM_ than for SGA_IG21_ and the aOR was only significant when using the STOPPAM reference (Fig. 3).

**Fig. 4:**
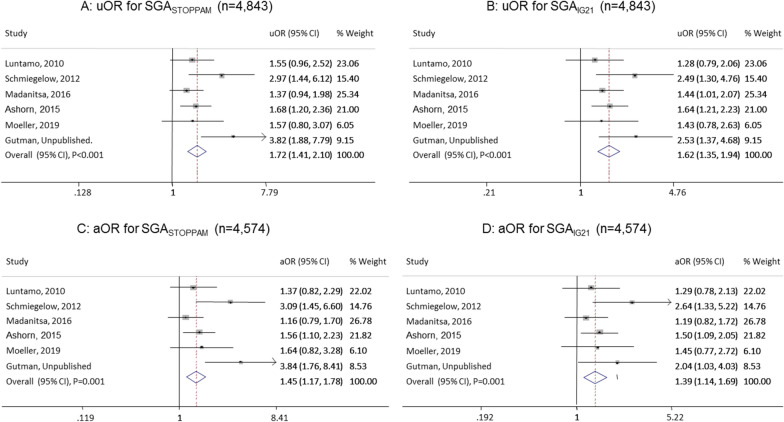
Association between SGA and malaria infection in the 1st and 2nd trimester vs. no malaria. Newborns of women having had mmalaria in the1st/2nd and 1st/2nd/3rd trimesters were pooled together but excluded if they had malaria in the 3^rd^ trimester only. Data from the study by Divala et al. were also excluded as malaria testing was only performed at enrolment and delivery. Panels **A** and **B** shows the unadjusted odds ratio (uOR) for small for gestational age (SGA) using STOPPAM (SGA_STOPPAM_) and Intergrowth-21 (SGA_IG21_) references while panels **C** and **D** shows the adjusted odds ratio (aOR), % Weights are from random effects analysis

The proportion of SGA was highest among newborns of women who had malaria in the 1st and/or 2nd trimester or in all three trimesters compared to malaria only in the 3rd trimester (Table 3). This was reflected in a strong association between MIP and SGA in these groups, both when using the STOPPAM reference (uOR 1.72 (1.41–2.10), p < 0.001; aOR 1.45 (1.17–1.78), p = 0.001) and the IG21 reference (uOR 1.62 (1.35–1.94), p < 0.001; aOR 1.39 (1.14–1.69), p = 0.001) (Fig. 4). Malaria infection during the 3rd trimester only was not significantly associated with SGA (Table 3).

**Table 3:** Association between malaria infection and small for gestational age, stratified by timing of infection

		STOPPAM reference							Intergrowth-21 reference						
Timing of infection^a^	N	SGA_STOPPAM_ n (%)	uOR	95% CI	P	aOR^b^	95% CI	P	SGA_IG21_ n (%)	uOR	95% CI	P	aOR^c^	95% CI	P
Never infected	3,371	420 (12.5)	1	–	–	1	–	–	579 (16.9)	1	–	–	1	–	–
1st/2nd trimester	1,214	205 (16.9)	1.60	1.31–1.96	< 0.001	1.35	1.09–1.66	0.006	250(20.6)	1.52	1.27–1.83	< 0.001	1.30	1.07–1.58	0.009
1st/2nd/3rd trimesters	258	52 (20.2)	2.37	1.65–3.41	< 0.001	1.73	1.19–2.52	0.004	56 (21.8)	2.18	1.54–3.10	< 0.001	1.68	1.16–2.43	0.006
3rd trimester only	646	102 (15.8)	1.47	1.14–1.91	0.003	1.28	0.98–1.67	0.07	108(16.7)	1.19	0.93–1.52	0.17	1.07	0.83–1.38	0.62

The pooled uOR (n = 5,489) and aOR (n = 5,208) were obtained using mixed effect model in the one stage individual participant data meta-analysis excluding a study by Divala et al. because malaria infection was only documented at inclusion and delivery

^a^stratified malaria into 3 groups, whereas in Fig. 4 we pooled together 1^st^/2^nd^ and 1^st^/2^nd^/3^rd^ and excluded 3^rd^ trimester only to allow for the forest plot, 1^st^ trimester: ≤ 13 weeks, 2^nd^ trimester: 14–27 weeks, 3^rd^ trimester: ≥ 28 weeks, *SGA*_*STOPPAM*_: Small for gestational age (SGA) using STOPPAM reference, *SGA*_*IG21*_: SGA using Intergrowth-21 reference, *uOR* unadjusted odds ratio, *aOR*: Adjusted odds ratio

^b^adjusted for body mass index, gravidity, gestational age at enrolment, HIV, and haemoglobin at enrolment

^c^adjusted for gestational age at delivery in addition to the confounders for aOR^b^, *CI* Confidence interval, malaria was defined as positive slide or malaria rapid test or polymerase chain reaction

### Other sensitivity analyses

The performance of the STOPPAM and IG21 references was also assessed after stratifying for malaria testing strategies, birthweight adjustment, GA ≤ 38 weeks, or HIV-seropositivity. Overall, similar results on the performance of the two references in detecting the association between MIP and SGA were obtained when excluding: studies using only one type of diagnostic test (RDT or PCR) or only testing symptomatic cases during antenatal visits, newborns with adjusted birthweight, newborns with GA ≤ 38 weeks, and HIV-seropositive. When solely defining malaria as microscopy blood smear positive, the effect of malaria was not statistically significant for neither SGA_STOPPAM_ nor SGA_IG21_ (Additional file 1: Table S6, Additional file 4: Fig. S3).

The association between malaria and SGA was not significant for neither SGA_IG21_-AGA_STOPPAM_ (aOR 0.90 (0.67–1.20), p = 0.47), nor SGA_STOPPAM_-AGA_IG21_ (aOR 1.02(0.62–1.69), p = 0.93) (Additional file 1: Table S7).

Finally, a two-stage individual participant data meta-analysis was applied, which yielded similar trends for the STOPPAM (uOR 1.57 (1.25–1.96); aOR 1.21 (0.99–1.43)) and the IG21 reference (uOR 1.39 (1.14–1.69); aOR 1.12 (0.92–1.31)) (Additional file 5: Fig. S4).

The trim and fill funnel plots revealed no strong evidence of small study effects for neither STOPPAM (p = 0.10) nor IG21 reference (p = 0.35). Similarly, the contour-enhanced funnel plot indicated the distribution of studies in both contours with small and large p-values (Additional file 6: Fig. S5).

## Discussion

Malaria can cause fetal growth restriction, preterm delivery, and SGA [2, 4, 5]. In this meta-analysis, the use of a local reference (STOPPAM) was more sensitive than a universal reference (IG21) in detecting a relationship between MIP and SGA. The effect was more pronounced among paucigravidae and following malaria infection in early pregnancy.

Malaria infection in early pregnancy is detrimental to fetal growth [2, 4, 5]. We likewise observed an increased risk of SGA after stratifying by early infections. The IG21 reference was less sensitive than STOPPAM in detecting an association between MIP and SGA. However, this was by far outweighed by the greater impact of malaria in early pregnancy, and the association between MIP and SGA_STOPPAM_ as well as SGA_IG21_ was statistically significant.

The association between MIP and SGA was similar for only paucigravidae compared to all gravidae. However, the aOR for IG21 was not statistically significant, reflecting the smaller aOR for SGA_IG21_ and the smaller sample size when including only paucigravidae. Similarly, the sample size was considerably reduced if malaria was only defined as microscopy positive. This resulted in statistically insignificant results for both references despite microscopy positivity normally being stronger associated with SGA than PCR positivity.

The IG21 classified 6.0% of full-term newborns as SGA who were AGA based on the STOPPAM reference. These newborns could be constitutionally small but healthy. Misclassification of SGA may diminish the ability to detect an effect of MIP on SGA. Indeed, the uOR and aOR were close to 1 in this sub-group.

The study also revealed that 1.6% of newborns (99/6,236) were SGA_STOPPAM_ but AGA_IG21_, with the majority being born preterm. The STOPPAM chart combines fetal weights and birthweights, whereas the IG21 chart is exclusively based on birthweight. Preterm newborns tend to be smaller than fetuses staying in the womb at the same GA [28, 47, 48]. This may explain the lower 10^th^ percentile for IG21 at preterm GA, resulting in a higher proportion of preterm newborns being classified as AGA on IG21. The association between MIP and SGA_STOPPAM_-AGA_IG21_ was statistically insignificant. This may partially be explained by the very small number of SGA_STOPPAM_-AGA_IG21_.

The findings from this meta-analysis support prior studies showing that an international reference may overestimate SGA in a high-risk population with increased exposure to multiple causes of poor fetal growth, including infections like malaria and poor nutrition [20, 49] while underestimating it in low-risk populations [50]. Several other studies [22, 23, 50, 51] found differences between local and global references. The results of this and previous studies [20–23, 49–51] strengthen the importance of having a reference chart derived from a local population, thereby accounting for geographical and ethnic differences. The FIGO position paper supports this approach [17].

The study findings have implications in research settings where the correct diagnosis of SGA is important to accurately estimate the effect of malaria interventions for improving pregnancy outcomes. When SGA is misclassified, particularly in randomized clinical trials using composite endpoints that include SGA, the treatment effect can be weakened, especially when the proportionate reduction of adverse pregnancy outcome is small.

The difference in the 10th percentile between the two references is critical. The study revealed a mean difference in the 10th percentiles between STOPPAM and IG21 of 133 g for boys and 123 g for girls at GA 40 + 0 weeks. This may affect a study’s ability to detect an impact on SGA given the relatively small difference in mean birthweight resulting from malaria interventions, such as insecticide-treated nets or IPTp with sulfadoxine-pyrimethamine (+ 2.5 to 3%) [52, 53].

It is important to determine which reference is the most representative for the study population. Both the IG21 and the STOPPAM reference were based on low-risk populations. Therefore, the IG21 and STOPPAM criteria were applied to limit the cohort to the healthy/low-risk population, expecting the prevalence of SGA to be close to 10% on a representative reference. However, SGA_IG21_ was 15% and 17% when applying the IG21 [43] and STOPPAM [28] criteria but only 9.6% and 11.5% for SGA_STOPPAM_, respectively. This indicates that the study population is more similar to the STOPPAM reference and the higher proportion of SGA when using IG21 is likely an overestimation.

There are differences in the construction of the two references, with the STOPPAM reference being a hybrid chart including fetal weights until GA 38 and IG21 being a pure birthweight chart. A levelling of growth was observed in late pregnancy on the STOPPAM reference. The Hadlock formula was used for estimating fetal weight, which may be slightly overestimated when applied in an African population [54]. This could explain the observed levelling. However, the flattening of the percentiles could also represent a true waning of growth as observed in other populations in late pregnancy [55, 56]. If restricting the analyses to newborns with GA > 38 weeks, and both references thereby constructed based on birthweights, a similar higher association between MIP and SGA_STOPPAM_ compared to MIP and SGA_IG21_ was observed. This supports the use of the STOPPAM reference despite the different methodology compared to IG21.

The strength of this analysis is the use of a large number of individual participants from a similar geographical area, all with GA estimated by ultrasound. The quality of birthweight was optimized by excluding newborns with unadjusted birthweight measured > 24 h post-delivery, twin deliveries, congenital malformations, and stillbirths. However, there are several limitations. First, the analysed studies used different methodologies for malaria diagnosis, with only one study including placenta histology and some studies using either only one type of malaria test or testing symptomatic cases only. This has a potential for misclassification bias. Except for the analyses on microscopy positivity (Additional file 1: Table S6), analysis based on a single test was not performed due to the small sample size that would have reduced the study power; however, the sensitivity analyses stratified by malaria diagnosis methodology gave robust results. This reasure that the results were not significantly affected. Furthermore, some studies adjusted birthweight measured > 24 h while others did not report the precise time of measurement, allowing for birthweight adjustment. However, excluding all adjusted birthweights did not alter the results, thereby justifying including them to obtain as large a sample size as possible.

The analysis excluded newborns missing information on sex, GA, or birthweight, and those with unadjusted birthweight measured > 24 h post-delivery. These accounted for a small proportion (12%) of total newborns and thus are unlikely to have led to selection bias. Furthermore, these newborns were not different from included newborns with respect to malaria exposure and the main confounders though there were some differences with respect to maternal age, gravidity, and BMI (Additional file 1: Table S8). The study compared two reference charts which were developed using different methodologies. Intergrowth-21 was only based on birthweight while STOPPAM is a hybrid of birthweight and fetal weight; the latter may be prone to errors, especially at a later GA with the expected uncertainty on the fetal weight of ± 10%.

Finally, the analysis conveniently included studies that were part of the IMPROVE Consortium (https://www.improve-consortium.org/). The study aim was not to get a precise estimate of the association between MIP and SGA through a systematic review including all possible studies, but rather to investigate the impact of using one reference versus another. Data were not obtained for three eligible studies. Therefore, it can’t be ruled out that this may have influenced the result, given the fewer studies for publication bias assessment.

## Conclusion

The higher birthweight percentiles at term for the IG21 reference may lead to an overestimation of SGA and an underestimation of malaria's potential impact on birthweight. When analysing the impact of malaria on pregnancy outcomes or trials of intervention to reduce malaria-associated fetal growth restriction, adding locally created reference charts to global references may be appropriate as they account for ethnic diversity and geographical differences.

## Supplementary Information

**Additional file 1: Table S1.** Comparison of the Intergrowth-21 and STOPPAM standard charts. **Table S2.** Quality of studies using Cochrane’s Risk of Bias 2 tool (ROB-2) for randomized trials. **Table S3.** Risk of Bias assessment by Newcastle scale for cohort studies. **Table S4.** Prevalence of small for gestational age by gestational age and birthweight. Table S5. Prevalence of small for gestational age when applying the inclusion criteria for each reference chart. **Table S6.** Sensitivity analysis for the association between malaria in pregnancy and SGA when using STOPPAM vs Intergrowth-21st reference. **Table S7.** association between malaria and small for gestational age for discordant groups. **Table S8.** Characteristics of analysed mother-newborn pairs vs excluded.

**Additional file 2: Figure S1.** Birth weight percentiles by sex and gestational age for STOPPAM vs. Intergrowth (IG21) references. The percentiles before and after 33 weeks for Intergrowth were merged, hence the bend in the 10th percentile.

**Additional file 3: Figure S2.** The birth weight z-scores comparing STOPPAM vs. Intergrowth (IG21) references. The dash line indicates the mean birthweight z-scores.

**Additional file 4: Figure S3.** Association between small for gestational age (SGA) and malaria in pregnancy excluding all HIV seropositive women. Panels A and B shows the unadjusted odds ratio (uOR) for SGA when using STOPPAM (SGA_STOPPAM_) and Intergrowth-21 (SGA_IG21_) references. Panels C and D shows the adjusted odds ratio (aOR).

**Additional file 5: Figure S4.** Two stage individual participant data meta-analysis on the association between malaria in pregnancy (MIP) and small for gestational age (SGA) using the STOPPAM (SGA_STOPPAM_) vs. the Intergrowth-21 reference (SGA_IG21_). uOR: unadjusted odds ratio in panels A and B, aOR: adjusted odds ratio in panels C and D controlling for body mass index, gravidity, gestational age at enrolment, HIV, and hemoglobin level at enrolment. In addition, adjusted for gestational age at delivery for SGA_IG21_, CI: confidence interval, malaria was defined as positive slide or positive malaria rapid test or positive polymerase chain reaction or positive placenta histology, % Weights are from random effects analysis.

**Additional file 6: Figure S5.** Funnel plots for meta-analysis. The closed dots indicate the observed studies, panel A indicate the trim and fill funnel plot for STOPPAM (P = 0.10) and panel B for Intergrowth-21 reference (P = 0.35). The contour enhanced funnel plot for STOPPAM (panel C) and Intergrowth-21 (panel D) show the distribution of studies in both the small and large p-values contours, hence no publication bias.

## Data Availability

Data used in this manuscript are available upon request subject to approval by the original authors.

## Abbreviations

SGA: Small for gestational age
AGA: Appropriate for gestational age
GA: Gestational age
MIP: Malaria in pregnancy
IG21: Intergrowth-21 birthweight reference chart
STOPPAM: Stop pregnancy associated malaria-fetal and birthweight reference chart
BMI: Body mass index
IMPROVE: Improving pregnancy outcome using intermittent preventive treatment of malaria in pregnancy
