# Supplementary material for: The choice of reference chart affects the strength of the association between malaria in pregnancy and small for gestational age: an individual participant data meta-analysis comparing the Intergrowth-21 with a Tanzanian birthweight chart
Source: Malar J. 2022 Oct 12;21:292. doi: 10.1186/s12936-022-04307-2 (PMC9559842; doi:10.1186/s12936-022-04307-2)
Supplement: Supplementary file 1 — Additional file 1: Table S1. Comparison of the Intergrowth-21 and STOPPAM standard charts. Table S2. Quality of studies using Cochrane’s Risk of Bias 2 tool (ROB-2) for randomized trials. Table S3. Risk of Bias assessment by Newcastle scale for cohort studies. Table S4. Prevalence of small for gestational age by gestational age and birthweight. Table S5. Prevalence of small for gestational age when applying the inclusion criteria for each reference chart. Table S6. Sensitivity analysis for the association between malaria in pregnancy and SGA when using STOPPAM vs Intergrowth-21st reference. Table S7. association between malaria and small for gestational age for discordant groups. Table S8. Characteristics of analysed mother-newborn pairs vs excluded. [file 12936_2022_4307_MOESM1_ESM.doc]

| **Table S1. Comparison of the Intergrowth-21 and STOPPAM standard charts** | | |
| --- | --- | --- |
|  | Intergrowth-21 | STOPPAM |
| Type of chart | Standard chart based solely on BW | Standard chart, hybrid of fetal & BW |
| Data collection period | From 2009-2014 | From 2008-2010 |
| Publication date | 2014 | 2012 |
| Settings | Urban | Rural and semi-urban |
| Population (n) | UK, Italy, Brazil, Oman, USA, China, India, and Kenya (20,486) | Tanzania (583) |
| Exclusion criteria | Age <18 or > 35 years | - |
|  | Maternal height<153 cm, BMI<18.5 or ≥30 Kg/m^2^ | Malnutrition defined as MUAC <23cm |
|  | History of smoking/alcohol use | History of current/previous smoking/alcohol use |
|  | Relevant past medical history with need of long term medication | Diabetes (pre-pregnancy diabetes or glucosuria and random blood sugar >11 mmol/L during pregnancy), asthma, and epilepsy |
|  | Hypertensive disorders, SBP>140 or DBP>90 mmHg. History of pre-eclampsia, eclampsia, HELLP syndrome or a related pregnancy-associated condition | Hypertension (SBP>140 or DBP>90 mmHg) or Pre-eclampsia (hypertension and proteinuria after GA 20) |
|  | Twins, previous baby <37 weeks or <2·5 kg or >4·5 kg, past/current miscarriage, stillbirth, or neonatal death | Twins, stillbirth, and preterm delivery <37 weeks in current pregnancy |
|  | Previous/current congenital malformation | Current severe congenital malformation |
|  | Positive urinalysis, STIs (syphilis, trichomoniasis), or malaria infection | Maternal Syphilis, HIV, or malaria infections |
|  | Clinically significant atypical red cell alloantibodies, diagnosis or treatment for anemia | Severe anemia (Hb<7 g/dL) at any time point during pregnancy using Sysmex hematological analyzer |
|  | Evidence of socio-economic constraints, exposure to chemicals or toxic substances, or very physically demanding activity. | - |
| Gestational age (GA) estimation | A combination of ultrasound (US) and LMP was used. CRL (Robinson) [29] was used if GA<14weeks, and BPD if GA14-24weeks. If a reliable LMP was known and GA 9+0 to 13+6w, LMP was used if the difference to US was <7 days; if >7 days US was used. | US was used until GA <=24+0. If GA was <11 weeks, a new estimation was performed within 2 months and the latter GA estimation used. CRL (Hadlock) [30] was used if GA<13+4, and HC (Chitty) [31] if GA 13+5 to 24+0 weeks. |
| Fetal weights (EFW) estimation | Serial US were performed by locally accredited ultrasonographers using identical US equipments in all sites. Serial US for EFW was done at 5-weekly (±1 week) intervals, i.e.14–18, 19–23, 24–28, 29–33, 34–38 and 39–42 weeks. EFW was calculated using INTERGROWTH-21 formula [32,33] | Serial US were done by a Danish medical doctor, and a locally trained midwife and medical doctor using the same US equipment. Serial US for EFW were done at 26-28, 30-32 and 36-38 weeks. EFW was obtained using the Hadlock algorithm [34]. |
| Measurement of BW | Newborn BW were obtained within 12 h of birth by using an electronic scale (Seca, Hangzhou, China). | Newborn BW were obtained within 24 hours of birth using a digital scale (ADE, 10g) and in few cases a Fazzini spring scale (50g). |
| Maternal age (years) | 28.4±3.9 | 27.1±6.2 |
| Maternal height (cm) | 161·8 ± 5·6 | 157.7±5.9 |
| Maternal weight (kg) | 61·3 ±8·6 | 55.8±9.4 |
| Maternal BMI (kg/m^2^) | 23·4 ± 2·9 | 22.1±3.6 |
| Gravidity (n (%)) | 12,996 (63·4%) were nulliparous | 99 (17.0%) were primigravidae |
| Years of education | 14·2 ± 3·0 | 383 (66.1) attained primary school |
| GA at inclusion (weeks) | 14·8 ±6·0 | 18.6±3.8 |
| Preterm (n (%)) | 1,136 (5.5%) | 0 (0.0%) |
| Term BW (Kg) | 3·3 ±0·5 | 3.2 ±0.5) |
| Low BW (n (%)) | 1,129 (5.5%) | 19 (3.8%) |
| Male newborns (n (%)) | 10,482 (51.2%) | 277 (47.5%) |
| BW: birthweight, BMI: body mass index, MUAC: mid-upper arm circumference, SBD: systolic blood pressure, DBP: diastolic blood pressure, STIs: sexually transmitted infections, CRL: crown ramp length, LMP: last menstrual period, BPD, biparietal diameter, HC: head circumference, AC: abdominal circumference, FL: femur length, the chi2 of proportions and ttest for the mean differences were all significant (p<0.05) except for male newborn.  References | | |

| **Table S2. Quality of studies using Cochrane’s Risk of Bias 2 tool (ROB-2) for randomized trials** | | | | | | | | |
| --- | --- | --- | --- | --- | --- | --- | --- | --- |
| Study | Sample size | Random sequence generation (Selection bias) | Allocation concealment (Selection bias) | Blinding of participant and personnel (Performance bias) | Missing outcome data (Attrition bias) | Blinding outcome measurement (Detection bias) | Selection of the reported result (Reporting bias) | Overall ROB |
| Luntamo, et al [45] | 1,327 | Low risk | Low risk | Low risk | Some concern:  3 (0.2%) missing BW, sex & GA.  1 (0.1%) missing BW & sex.  121 (9.1%) had BW>24 hrs unadjusted. | Low risk | Low risk | Low |
| Ashorn, et al [37] | 1,403 | Low risk | Low risk | Low risk | High risk^a^:  84 (6.0%) missing BW, sex & GA.  96 (6.9%) missing BW.  2 (0.1%) missing sex.  352(25.1%) had BW>24 hrs adjusted. | Low risk | Low risk: | High |
| Madanitsa, et al [38] | 1,873 | Low risk | Low risk | Some concern: Open label | Some concern^b^:  121 (6.5%) missing BW, sex & GA.  90 (4.8%) missing BW & sex.  77 (4.1%) had BW>24 hrs adjusted. | Some concern:  No blinding | Low risk | Low |
| Divala, et al [41] | 908 | Low risk | Low risk | Some concern: Open label | High risk^c^:  79 (8.7%) missing BW, sex & GA  15 (1.7%) missing BW.  0 (0.0%) had BW>24hrs. | Low risk | Low risk | High |
| Gutman, unpublished | 601 | Low risk | Low risk | Some concern: Open label | High risk^d^:  48 (8.0%) missing BW, sex & GA  10 (1.7%) missing BW  2(0.5%) missing sex  34 (5.7%) had BW >24 hours unadjusted. | Low risk:  blinded for malaria microscopy | Low risk | High |
| ROB: Risk of bias, studies were regarded as high quality if all pillars were of low ROB or if there were some concerns but the risk was unlikely to affect the birthweight (BW) measurement which is the outcome of this analysis while the presence of high risk in any of the pillar was regarded as poor quality, mRDT: malaria rapid diagnostic test, PCR: polymerase chain reaction, GA: gestational age, ANC: antenatal care, IST-DP: intermittent screening and treatment with dihydroartemisinin-piperaquine, IPT-SP: intermittent preventive treatment of malaria in pregnancy using sulfadoxine-pyrimethamine, ^a^ Used only mRDT for malaria testing, ^b^ Tested for malaria using microscope or PCR or mRDT in IST-DP arm and for IPTp-SP arm if symptomatic, ^c^ Provided malaria data for enrolment and delivery only, ^d^ All women had malaria testing at enrollment and delivery irrespective of symptoms except for the follow-up visits where only symptomatic cases were tested for malaria. | | | | | | | | |

| **Table S3. Risk of Bias assessment by Newcastle scale for cohort studies** | | | | | | | | | |
| --- | --- | --- | --- | --- | --- | --- | --- | --- | --- |
| Study | Sample size | Representativeness of the exposed | Selection of the non-exposed | Ascertainment of exposure | Demonstration that outcome of interest was not present at start of study | Control for confounders | Assessment of outcome | Adequacy of follow up | Overall ROB |
| Schmiegelow, et al [28] | 924 | Representative as women were enrolled consecutively during antenatal visits | No selection, consecutive enrolment, malaria was not an inclusion or exclusion criteria. | Malaria exposure was ascertained through repeated tests at fixed time points from enrolment to delivery | Outcome was not present at enrolment as the women were followed from early pregnancy | Potential confounders were measured, assessed and reported | BW measured twice and used the average, or thrice if the difference was >50g | 45(4.9%) missing BW.  1 (0.1%) missing sex. 4 (0.4%) missing BW and GA.  80 (8.7%) had BW >24 hours unadjusted | Low |
| Moeller, et al [44] | 427 | Women were enrolled consecutively during antenatal visits | No selection, consecutive enrolment, malaria was not an inclusion or exclusion criteria | Malaria exposure was ascertained through repeated tests at fixed time points from enrolment to delivery | Outcome was not present at enrolment as the women were followed from early pregnancy | Potential confounders were measured, assessed and reported | BW measured twice and used the average, or thrice if the difference was >50g | 14(3.3%) missing BW.  0 (0.0%) missing sex. 0 (0.0%) missing GA. 10 (2.3%) had BW >24 hours unadjusted. | Low |
| ROB: Risk of bias, Studies were determined as Low ROB if retention rate was >75%, 80% of birthweights (BW) were measured within 24 hours of delivery, and a clear description of measurement of outcome (BW), exposures (malaria) and important confounders, GA: gestational age, BW: birthweight. | | | | | | | | | |

| **Table S4. Prevalence of small for gestational age by gestational age and birthweight** | | | | | | | |
| --- | --- | --- | --- | --- | --- | --- | --- |
|  |  | Preterm (N=616) | | | Term (N=5,620) | | |
| Reference |  | n | LBW (n=259) | NBW (n=357) | n | LBW (n=385) | NBW (n=5,235) |
| SGA_IG21_ | Yes | 43 | 43 (7.0) | 0 (0.0) | 1,081 | 350 (6.2) | 731 (13.0) |
|  | No | 573 | 216 (35.0) | 357(58.0) | 4,539 | 35 (0.6) | 4,504 (80.1) |
| SGA_STOPPSM_ | Yes | 95 | 95 (15.4) | 0 (0.0) | 792 | 362 (6.4) | 430 (7.7) |
|  | No | 521 | 164 (26.6) | 357(58.0) | 4,828 | 23 (0.4) | 4,805 (85.5) |
| Preterm defined as gestational age (GA) <37 weeks and Term as GA ≥37 weeks, LBW: low birthweight (<2500g), NBW: normal birth weight (≥2500g), SGA_IG21_; small for GA based on the intergrowth-21 reference, SGA_STOPPSM_: small for GA based on the STOPPAM reference chart | | | | | | | |

| **Table S5. Prevalence of small for gestational age when applying the inclusion criteria for each reference chart** | | | | | | |
| --- | --- | --- | --- | --- | --- | --- |
| Criteria ^a^ | N | SGA_IG21_ | | SGA_STOPPAM_ | | p |
|  |  | n (%) | 95% CI | n (%) | 95% CI |  |
| Intergrowth-21 ^b^ | 1,729 | 257 (14.9) | 13.3-16.7 | 166 (9.6) | 8.3-11.1 | <0.001 |
| STOPPAM ^c^ | 3,171 | 546 (17.2) | 15.9-18.6 | 366 (11.5) | 10.5-12.7 | <0.001 |
| ^a^ data not available for some risk factors including Diabetes, epilepsy, pre-eclampsia, hypertension, smoking/alcohol use, SGA_IG21_: prevalence of small for gestational age (SGA) when using the intergrowth-21 reference (IG21), SGA_STOPPAM_: prevalence of small for gestational age (SGA) based on the STOPPAM reference, ^b^ excluded all women/newborns with risk factors based on available data: twins, stillbirth, congenital malformation, body mass index >=30 or <18.5 kg/m^2^, hemoglobin at enrolment <7g/dL, HIV seropositive, syphilis, age<18 or >35 years, height<153 cm, and any positive malaria test, ^c^ excluded all women and newborns with risk factors based on available data: twins, stillbirth, congenital malformation, mid upper arm circumference at enrolment <23 cm, hemoglobin at enrolment <7g/dl, HIV seropositive, syphilis, any positive malaria test, and preterm newborns, p: p-values by chi^2^ comparing between SGA_IG21_ and SGA_STOPPAM_, CI: confidence interval | | | | | | |

| **Table S6. Sensitivity analysis for the association between malaria in pregnancy and SGA when using STOPPAM vs. Intergrowth-21^st^ reference** | | | | | | | | | | |
| --- | --- | --- | --- | --- | --- | --- | --- | --- | --- | --- |
| **Criteria applied** | **Reference** | **Luntamo *et al*** [45] | **Schmiegelow *et al*** [28] | **Madanitsa *et al*** [38] | **Ashorn *et al*** [37] | **Divala *et al*** [41] | **Moeller *et al*** [44] | **Gutman *et al Unpublished*** | **Overall uOR**  **STOPPAM**  **vs IG21** | **Overall aOR**  **STOPPAM**  **vs IG21** |
| Malaria defined by positive mRDT and/or slide and/or PCR and/or placenta histology excluding a study relying only on mRDT | STOPPAM | 35/168  vs  150/1,005 | 11/52  vs  67/700 | 176/ 1,214 vs  43/393 | NA | 17/114  vs  90/632 | 29/143  vs  40/232 | 20/141  vs  16/332 | **1.50(1.22-1.83**) | **1.30(1.05-1.60)** |
|  | IG21 | 35/168  vs  177/1,005 | 15/52  vs  114/700 | 187/1,214  vs  46/393 | NA | 19/114  vs  120/632 | 40/143  vs  59/232 | 24/141  vs  28/332 | **1.32(1.10-1.60)** | 1.15(0.94-1.40) |
| Malaria defined by positive mRDT and/or slide and/or PCR and/or placenta histology excluding a study relying only on PCR | STOPPAM | 35/168  vs  150/1,005 | 11/52  vs  67/700 | 176/ 1,214 vs  43/393 | 88/400  vs  104/709 | NA | 29/143  vs  40/232 | 20/141  vs  16/332 | **1.60(1.34-1.92)** | **1.35(1.11-1.63**) |
|  | IG21 | 35/168  vs  177/1,005 | 15/52  vs  114/700 | 187/1,214  vs  46/393 | 113/400  vs  146/709 | NA | 40/143  vs  59/232 | 24/141  vs  28/332 | **1.37(1.16-1.61**) | **1.25(1.04-1.49)** |
| Systematic tested for malaria, excluding a study only testing symptomatic cases | STOPPAM | 35/168  vs  150/1,005 | 11/52  vs  67/700 | 176/ 1,214 vs  43/393 | 88/400  vs  104/709 | 17/114  vs  90/632 | 29/143  vs  40/232 | NA | **1.46(1.23-1.75)** | **1.24(1.03-1.50)** |
|  | IG21 | 35/168  vs  177/1,005 | 15/52  vs  114/700 | 187/1,214  vs  46/393 | 113/400  vs  146/709 | 19/114  vs  120/632 | 40/143  vs  59/232 | NA | **1.33(1.13-1.57)** | 1.16(0.97-1.38) |
| All birthweight directly measured, excluding newborns with adjusted BW | STOPPAM | 35/168  vs  150/1,005 | 11/52  vs  67/700 | 169/1,158  vs  42/373 | 69/288  vs  72/483 | 17/114  vs  90/632 | 29/143  vs  40/232 | 20/141  vs  16/332 | **1.55(1.30-1.86)** | **1.33(1.11-1.61)** |
|  | IG21 | 35/168  vs  177/1,005 | 15/52  vs  114/700 | 176/1,158  vs  46/373 | 89/288  vs  105/483 | 19/114  vs  120/632 | 40/143  vs  59/232 | 24/141  vs  28/332 | **1.37 (1.16- 1.61)** | **1.19(1.00-1.42)** |
| Malaria defined as microscopy positive, excluding malaria cases only positive on mRDT or PCR positive or positive placenta histology | STOPPAM | 30/136  vs  150/1,005 | 5/33  vs  67/700 | 74/ 452  vs  43/393 | ND | ND | 15/90  vs  40/232 | 1/10  vs  15/326 | **1.48(1.14-1.92)** | 1.27(0.98-1.68) |
|  | IG21 | 31/136  vs  177/1,005 | 9/33  vs  114/700 | 84/ 452  vs  46/393 | ND | ND | 20/90  vs  59/232 | 2/10  vs  28/326 | **1.43(1.13-1.82)** | 1.27(0.98-1.64) |
| Excluding newborns with GA<=38weeks^a^ | STOPPAM | 21/94  vs  98/608 | 10/49  vs  54/649 | 78/572  vs  21/201 | 65/304  vs  76/548 | 15/81  vs  57/456 | 23/127  vs  33/209 | 15/72  vs  6/174 | **1.69(1.37-2.09)** | **1.44(1.16-1.79)** |
|  | IG21 | 25/94  vs  129/608 | 14/49  vs  105/649 | 127/572  vs  34/201 | 93/304  vs  124/548 | 17/81  vs  96/456 | 35/127  vs  54/209 | 20/72  vs  23/174 | **1.43(1.19-1.71)** | 1.20(0.99-1.45) |
| Numbers for each study represent number with SGA/malaria positive vs SGA/malaria negative for SGA_STOPPAM_ (small for gestational age using STOPPAM reference) and SGA_IG21_ (SGA using Intergrowth-21 reference), mRDT: malaria rapid diagnostic test, PCR: polymerase chain reaction, uOR: unadjusted odds ratio, aOR: adjusted odds ratio, NA: not applicable, ND: not done, in bold: significant uOR and/or aOR, ^a^the STOPPAM reference was based o fetal weight until 38 weeks, thereafter birthweight was used similar to IG21. | | | | | | | | | | |

| **Table S7: association between malaria and small for gestational age for discordant groups** | | | | | | | |
| --- | --- | --- | --- | --- | --- | --- | --- |
| SGA sub-groups | | Malaria | No malaria | uOR (95% CI) | P | aOR (95% CI) | P |
| SGA_IG21_/ AGA_STOPPAM_ | Yes | 107 | 229 | 0.99 (0.75-1.30), | 0.92 | 0.90 (0.67-1.20) | 0.47 |
|  | No | 1,749 | 3,264 | 1 | - | 1 | - |
| SGA_STOPPAM_/ AGA_IG21_ | yes | 50 | 49 | 1.17 (0.73-1.90), | 0.52 | 1.02(0.62-1.69) | 0.93 |
|  | No | 1,749 | 3,264 | 1 | - | 1 | - |
| SGA_IG21_/ AGA_IG21_: small/adequate for gestational age (GA) based on the intergrowth-21 reference, SGA_STOPPSM_/ AGA_STOPPAM_: small /adequate for GA based on the STOPPAM reference chart, uOR: unadjusted odds ratio, aOR: adjusted odds ratio, CI: confidence interval. | | | | | | | |

| **Table S8. Characteristics of analyzed mother-newborn pairs vs excluded*** | | | | | |
| --- | --- | --- | --- | --- | --- |
| **Characteristics** | **Analyzed (N=6,236)** | | **Excluded (n=931)** | | **P-Value** |
|  | N | n (%)/mean (SD)/median (IQR) | N | n (%)/mean (SD)/median (IQR) |  |
| Age (years) ^a^ | 6,231 | 19 (23-28) | 757 | 19(22-26) | <0.001 |
| MUAC (cm) ^b^ | 3,862 | 26.1 (2.9) | 426 | 26.4(2.9) | 0.08 |
| Maternal Weight | 6,218 | 55.2(8.8) | 875 | 55.8(8.7) | 0.05 |
| Maternal Height | 6,176 | 155.7(5.7) | 854 | 155.8447(5.7) | 0.54 |
| Maternal BMI (kg/m2) ^a^ | 6,161 | 22.8 ( 3.2) | 742 | 23.1(3.2) | 0.01 |
| Paucigravidae | 6,229 | 3,364 (54.0%) | 754 | 471(62.5%) | <0.001 |
| ITN use | 6,232 | 5,841 (86.4) | 581 | 477 (82.1%) | <0.01 |
| # ANC visits ^a^ | 5,099 | 4(4-6) | 492 | 3(1-4) | <0.001 |
| # of IPTp doses ^a^ | 3,907 | 2 (2-4) | 306 | 1(1.5-3) | <0.001 |
| HIV positivity | 6,045 | 340 (5.6%) | 706 | 41(5.8%) | 0.84 |
| Syphilis positive | 1,528 | 60 (3.9%) | 127 | 3 (2.4%) | 0.38 |
| Hb (g/dl) ^b^ |  |  |  |  |  |
| Enrolment | 6,224 | 11.1 (1.6) | 756 | 11.1(1.6) | 0.34 |
| Delivery | 1,559 | 11.4 (1.8) | 18 | 11.9(1.9) | 0.06 |
| Malaria prevalence | 6,235 | 2,232 (35.8%) | 757 | 259(34.2%) | 0.39 |
| GA delivery (days) ^a^ | 6,236 | 39 (38-40) | 392 | 39 (37-41) | 0.92 |
| * excluded newborns due to missing birthweight or sex or GA or GA>42+6 weeks or unadjusted birthweight measured>24 hours post-delivery,  ^a^ median (interquartile rage), ^b^ mean (standard deviation), MUAC: mid upper arm circumference, BMI: body mass index, ITN: treated bednet,  ANC: antenatal care, IPTp: intermittent preventive testament of malaria in pregnancy, GA: gestational age. P-value obtained by chi^2^ for proportion,  ttest for the mean difference and ranksum for the median. | | | | | |
