# Supplementary figures and images for: The choice of reference chart affects the strength of the association between malaria in pregnancy and small for gestational age: an individual participant data meta-analysis comparing the Intergrowth-21 with a Tanzanian birthweight chart
Source: Malar J. 2022 Oct 12;21:292. doi: 10.1186/s12936-022-04307-2 (PMC9559842; doi:10.1186/s12936-022-04307-2)

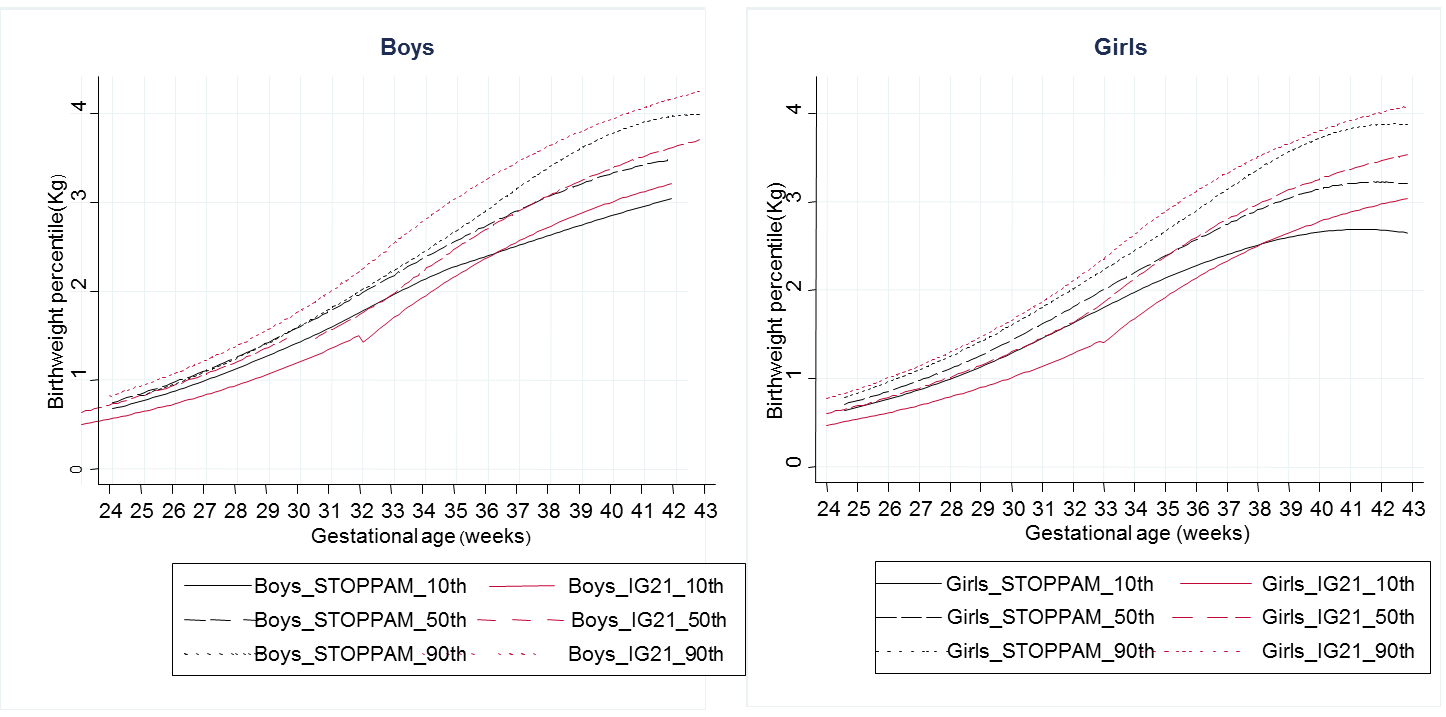

Supplement: Supplementary file 2 — Additional file 2: Figure S1. Birth weight percentiles by sex and gestational age for STOPPAM vs. Intergrowth (IG21) references. The percentiles before and after 33 weeks for Intergrowth were merged, hence the bend in the 10th percentile. [file 12936_2022_4307_MOESM2_ESM.tif]

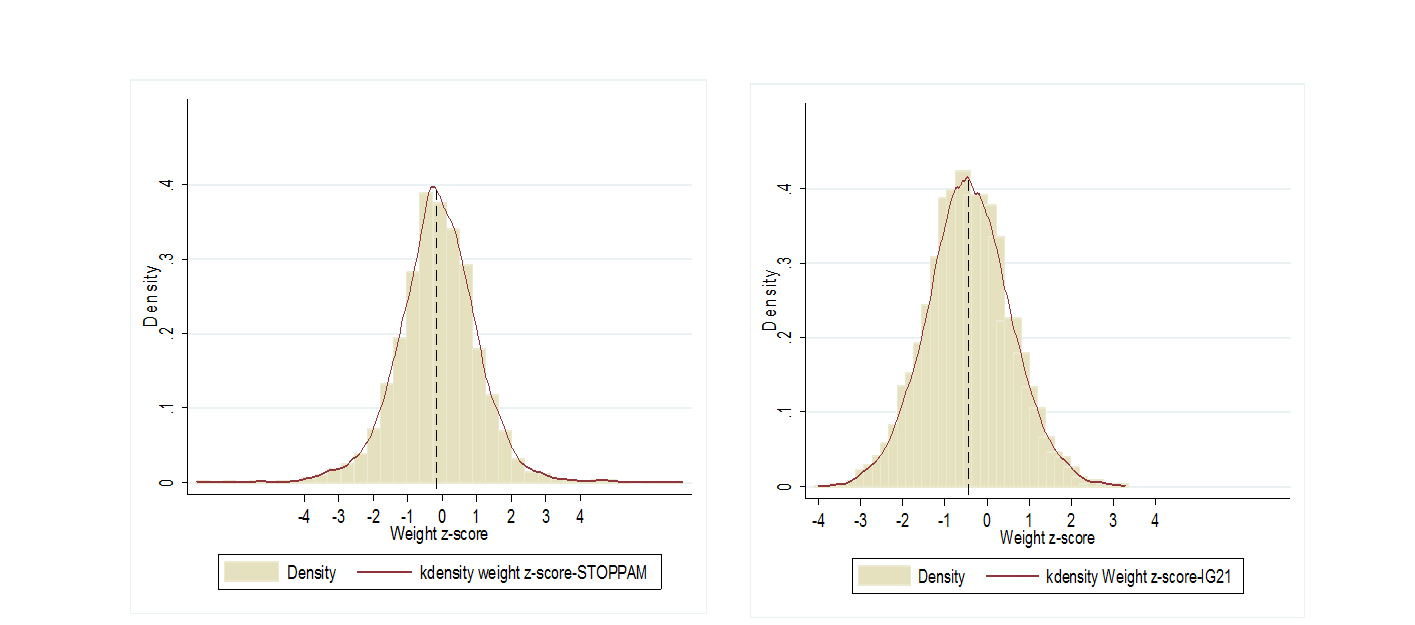

Supplement: Supplementary file 3 — Additional file 3: Figure S2. The birth weight z-scores comparing STOPPAM vs. Intergrowth (IG21) references. The dash line indicates the mean birthweight z-scores. [file 12936_2022_4307_MOESM3_ESM.tif]

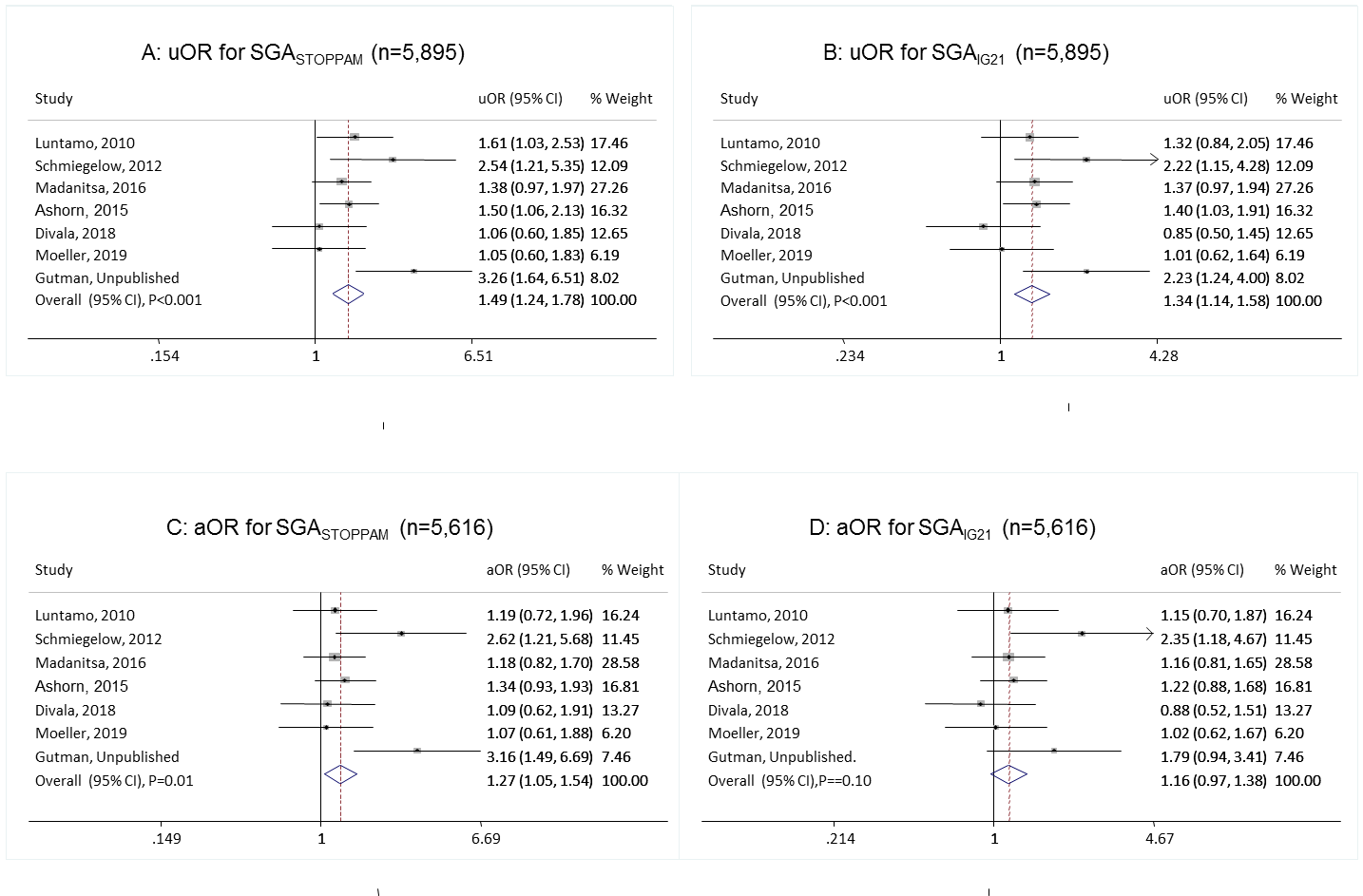

Supplement: Supplementary file 4 — Additional file 4: Figure S3. Association between small for gestational age (SGA) and malaria in pregnancy excluding all HIV seropositive women. Panels A and B shows the unadjusted odds ratio (uOR) for SGA when using STOPPAM (SGASTOPPAM) and Intergrowth-21 (SGAIG21) references. Panels C and D shows the adjusted odds ratio (aOR). [file 12936_2022_4307_MOESM4_ESM.tif]

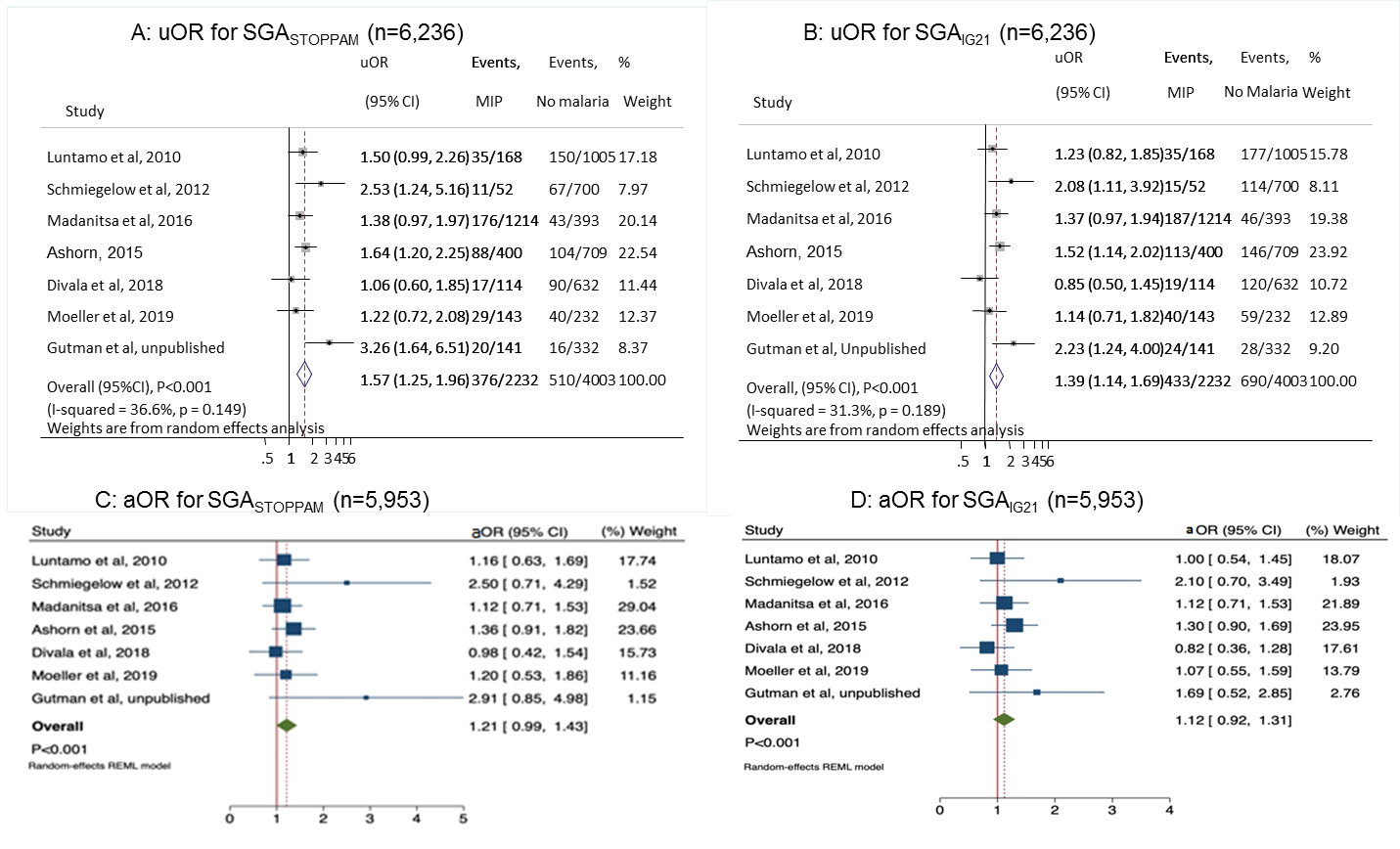

Supplement: Supplementary file 5 — Additional file 5: Figure S4. Two stage individual participant data meta-analysis on the association between malaria in pregnancy (MIP) and small for gestational age (SGA) using the STOPPAM (SGASTOPPAM) vs. the Intergrowth-21 reference (SGAIG21). uOR: unadjusted odds ratio in panels A and B, aOR: adjusted odds ratio in panels C and D controlling for body mass index, gravidity, gestational age at enrolment, HIV, and hemoglobin level at enrolment. In addition, adjusted for gestational age at delivery for SGAIG21, CI: confidence interval, malaria was defined as positive slide or positive malaria rapid test or positive polymerase chain reaction or positive placenta histology, % Weights are from random effects analysis. [file 12936_2022_4307_MOESM5_ESM.tif]

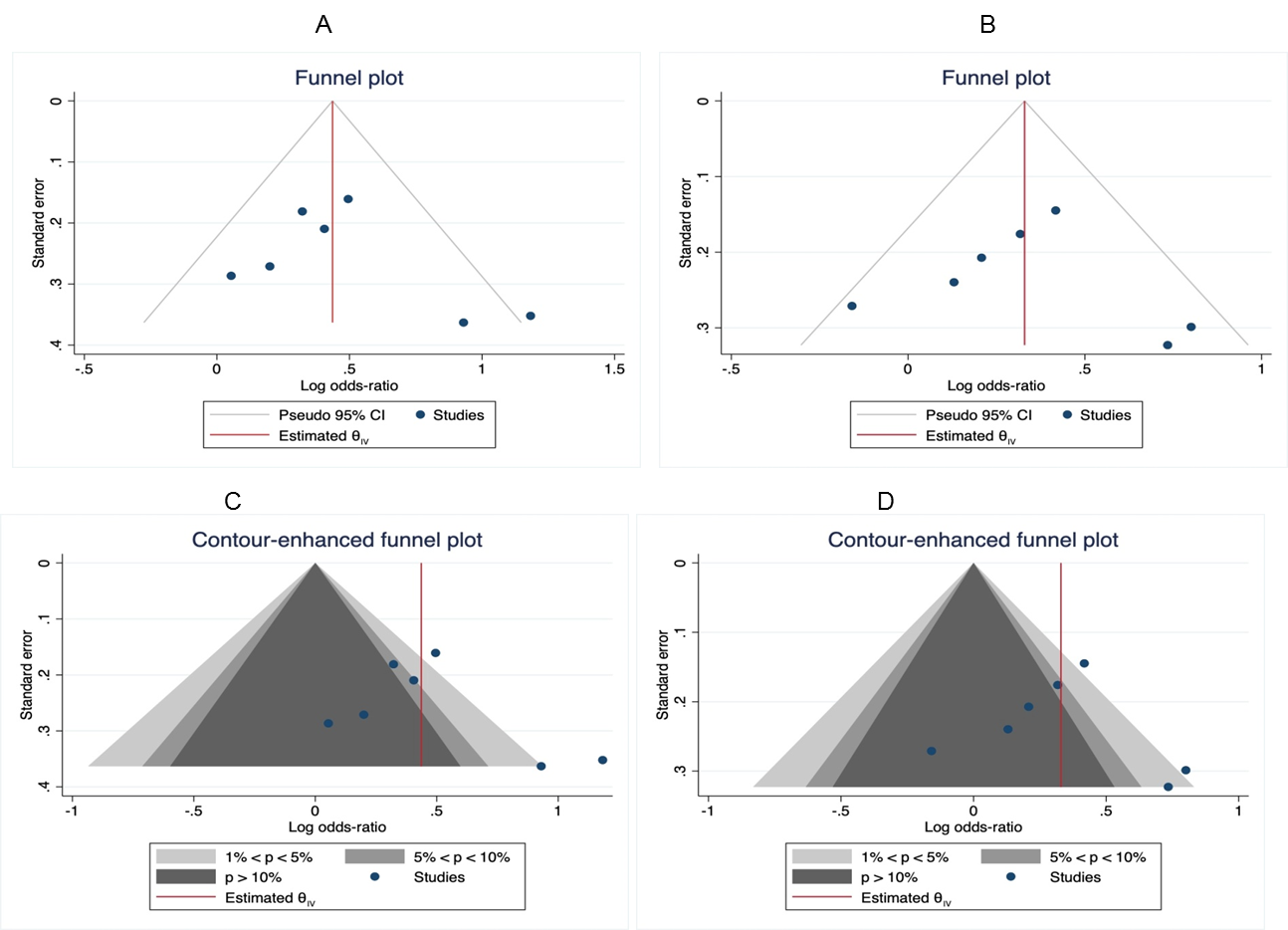

Supplement: Supplementary file 6 — Additional file 6: Figure S5. Funnel plots for meta-analysis. The closed dots indicate the observed studies, panel A indicate the trim and fill funnel plot for STOPPAM (P = 0.10) and panel B for Intergrowth-21 reference (P = 0.35). The contour enhanced funnel plot for STOPPAM (panel C) and Intergrowth-21 (panel D) show the distribution of studies in both the small and large p-values contours, hence no publication bias. [file 12936_2022_4307_MOESM6_ESM.tif]
